# High relative environmental humidity is associated with diabetes among elders living in Mediterranean islands

**DOI:** 10.1186/2251-6581-13-25

**Published:** 2014-02-03

**Authors:** Stefanos Tyrovolas, Christos Chalkias, Marianthi Morena, Kleomenis Kalogeropoulos, Nikos Tsakountakis, Akis Zeimbekis, Efthimios Gotsis, George Metallinos, Vassiliki Bountziouka, Christos Lionis, Evangelos Polychronopoulos, Demosthenes Panagiotakos

**Affiliations:** 1 Department of Nutrition and Dietetics, Harokopio University, 46 Paleon Polemiston St., Glyfada, 166 74, Attica, Athens, Greece; 2 Department of Geography, Harokopio University, Athens, Greece; 3 Clinic of Social and Family Medicine, School of Medicine, University of Crete, Heraklion, Greece; 4 Health Center of Kalloni, General Hospital of Mitilini, Mitilini, Greece; 5 Parc Sanitari Sant Joan de Déu, Fundació Sant Joan de Déu, CIBERSAM, Universitat de Barcelona, Dr Antoni Pujades, 42, 08830, Sant Boi de Llobregat, Barcelona, Spain

**Keywords:** Relative humidity, Temperature, Diabetes, Elderly, Diet, Mediterranean islands

## Abstract

**Background:**

Climate variation has long been studied in relation to human health. The aim of the present work was to evaluate the relationship between environmental humidity, and air temperature with the prevalence of diabetes, among elderly islanders.

**Methods:**

During 2005–2011, 1959 elderly (aged 65 to 100 years) individuals from 13 Mediterranean islands were enrolled. Socio-demographic, clinical and lifestyle factors were assessed using standard procedures. Diabetes was defined as fasting blood glucose levels > 125 mg/dl. Relative environmental humidity was measured as a percentage of air moisture and mean daily temperature in degrees Celsius.

**Results:**

For the present analysis 713 men (74 ± 7 years) and 596 women (73 ± 7 years) with complete data were studied; 27% of both men and women had diabetes. The prevalence of diabetes was 42% in the elders living in high relative humidity areas (i.e., >70%) as compared with 24% among those living at low relative humidity residential areas (p < 0.001). After adjusting for age, sex and mean temperature, an increase in the area’s relative humidity by 1 degree, increased the likelihood of having diabetes by 12% (OR = 1.12, 95% CI 1.05 to 1.20). No significant association was observed between mean temperature and diabetes (OR = 0.97, 95% CI 0.74, 1.26).

**Conclusions:**

A considerable proportion of elderly, especially those living in high relative humidity areas, had diabetes. Further research is needed to confirm this observation and to understand the underlying mechanisms.

## Introduction

Worldwide 347 million of people have diabetes [1]. There are a variety of lifestyle and clinical factors related to the development of diabetes [2-4]. Diabetes has been associated with increased risk for the development of cardiovascular disease. Furthermore it is a major cause of stroke and other chronic diseases [5]. The adoption of unhealthy dietary habits and the sedentary life are associated with the promotion of diabetes [6-8]. Therefore, several studies suggested that lifestyle modifications (i.e., healthy nutrition, nutritional education and exercise can minimize the risk of developing this chronic disease [9]. A recent study from the metropolitan area of Greece, reported that the prevalence of diabetes was 25% and 31% among the older males and females participants [10], while other studies from insular areas indicated that the prevalence of the disease was over 20% [11,12]. A variety of bio-clinical, lifestyle-related and environmental factors have contributed to the increase of diabetes at alarming rates.

Climate variation [13-15] and air pollution [16] has long been studied in relation to human health. It has been suggested that variations in environmental humidity and temperature are associated with myocardial infarction deaths [17] and hospitals admissions for acute coronary syndromes [18]. The relationship between temperature and cardiovascular events is consistent [19,20] while the association with humidity appeared to be inconsistent [18,19]. According to recent results, a 1% increase of relative environmental humidity is a result for 0.7% increase in acute coronary syndromes admissions [21]. Until now, none of these studies have proposed a possible explanation between high relative humidity and cardiovascular events and the pathophysiologic relation mechanism seems to be unknown. The scientific interest has turned to the association between cardiovascular disease (CVD) mortality rates [17,18] and climate variation (mostly with temperature [22]), but only a limited number of studies had actually investigated the effect of climate conditions to older population’s morbidity from cardiovascular factors such as obesity and diabetes.

Diabetes is a clinical condition, often asymptomatic and with high prevalence in older adults. Given the lack of data regarding the effect of environmental factors, and particularly climatological, on the prevalence of diabetes, the aim of the present work was to evaluate the association between relative humidity and mean air temperature, with the prevalence of diabetes, among elders (> 65 years old) that are lifelong inhabitants of Mediterranean islands.

## Methodology

### The MEDIS study’s sample

This work is under the context of the MEDIS Study [12]. During 2005–2011, a population-based, multi-stage, convenience sampling [(i.e., three age group levels (65–75, 75–85, 85±) and both genders)] was performed to voluntarily enroll elders from 13 Mediterranean islands [23]: Malta (n = 250), Republic of Cyprus (n = 300) and the Greek islands of Mitilini (n = 142), Samothraki (n = 100), Cephalonia (n = 115), Crete (n = 131), Corfu (n = 149), Limnos (n = 150), Ikaria (n = 76), Syros (n = 151), Naxos (n = 145), Zakynthos (n = 103) and Salamina (n = 147). The participation rate varied from 75% to 89% between the islands. The number of enrolled participants is adequate achieving statistical power 0.85 or greater for testing two-sided hypothesis for odds ratios for the main investigated variables (i.e., diet score, physical activity) equal to 1.20 at 0.05 type I error rate. For the present analysis and since geographical data from Malta and Republic of Cyprus were not available, information from 713 men, aged 74 ± 7 years and 596 women, aged 73 ± 7 years was studied. Individuals were not included in the study if they resided in assisted-living centers, or had a clinical history of CVD or cancer, or they had left the island for a considerable period of time during their life (i.e. >5 years). A group of health scientists (physicians, dietitians, public health nutritionists and nurses) with experience in field investigation collected all the required information using a quantitative questionnaire and standard procedures [12,23].

The study followed the ethical considerations provided by the World Medical Association (52^nd^ WMA General Assembly, Edinburgh, Scotland, October 2000). The Institutional Ethics Board of Harokopio University approved the study design (16/19-12-2006). Participants were informed about the aims and procedures of the study and gave their consent prior to being interviewed.

### Evaluation of clinical characteristics

As already has been presented in previous reports from the MEDIS study [12,23], all the measurements taken in the different study centers were standardized. Weight and height were measured using standard procedures and body mass index (BMI) in kg/m^2^ was calculated. Overweight was defined as BMI between 25 and 29.9 Kg/m^2^, while obesity was defined as BMI > 29.9 Kg/m^2^. Moreover, waist circumference in cm was measured in the middle between the 12^th^ rib and the iliac crest and hip circumference in cm was measured around the buttocks. Central fat was defined as waist circumference greater than 102 cm for men and 88 cm for women. Diabetes was determined by fasting plasma glucose tests greater than 125 mg/dl, or use of under anti diabetic drug or insulin. Participants who had blood pressure levels ≥140/90 mmHg or used antihypertensive medications were classified as hypertensive. Fasting blood lipids levels were also recorded and hypercholesterolemia was defined as total serum cholesterol levels >200 mg/dL or the use of lipid-lowering agents, according to the NCEP ATPIII guidelines [24]. The coefficient of variation for the blood measurements was less than 5%.

### Climatological evaluation

In this work, the mean percent of relative environmental humidity and the mean rates of temperature (in degrees of Celsius) during the past 5 years before sampling, were recorded using data from the National Meteorological Service [25]. Areas with relative humidity above 70% were considered as high environmental humidity and the rest as low humidity areas. This comparison was based on the assumption that relative humidity between 70-80% is considered to be high and very close to tropical conditions [26].

### Evaluation of dietary habits, socio-demographic and other lifestyle characteristics

As already has been presented in previous reports from the MEDIS study [12,23] the dietary habits were assessed through a semi-quantitative, validated and reproducible food-frequency questionnaire [27]. To evaluate the level of adherence to the Mediterranean diet, the MedDietScore (possible range 0–55) was used [28]. Higher values for this diet score indicate greater adherence to the Mediterranean diet. Furthermore the median value of the MedDietScore, i.e., 34/55 was used a cutoff to classify participants as away or moderate/close adheres. Participants were also encouraged to report the duration of their dietary habits (i.e. the number of years for which they had followed this dietary pattern). Moreover, various socio-demographic characteristics, such as age, gender, annual income and lifestyle characteristics, like smoking habits and physical activity status were also collected. Current smokers were defined as smokers at the time of the interview. Former smokers were defined as those who previously smoked, but had not done so for a year or more. The remaining participants were defined as occasional or non-current smokers. Physical activity was evaluated in MET-minutes per week, using the shortened, translated and validated into Greek, version of the self-reported International Physical Activity Questionnaire (IPAQ) [29]. Frequency (times per week), duration (minutes per time) and intensity of physical activity during sports, occupation and/or free-time activities were assessed. Participants were classified as inactive, minimally active and HEPA active (health enhancing physical activity; a high active category).

### Statistical analysis

Continuous variables were presented as mean ± standard deviation (SD), and categorical variables as frequencies. Comparisons of normally distributed continuous variables (i.e., age, school years, MedDietScore, Body Mass Index, systolic and diastolic blood pressure, fasting blood glucose levels, total cholesterol levels) between groups were performed using Student’s t-test. Spearman rho coefficient was applied to evaluate the correlation between mean relative humidity, mean temperature and physical activity, the MedDietScore of the participants. Normality was tested using P-P plots. Independence between categorical variables was tested using the chi-square criterion. Additive logistic regression models were used to evaluate the association between participants’ characteristics (i.e., age, sex, area’s relative humidity and mean temperature, physical activity, adherence to the Mediterranean diet, smoking habits, living conditions obesity, hypertension, hypercholesterolemia) and presence of diabetes. Results are expressed as odds ratios and the 95% confidence intervals. Deviance residuals and Hosmer-Lemeshow criterion evaluated models’ goodness-of-fit. P-value <0.05 was considered to be statistically significant. SPSS software (version 18) was used for all calculations (SPSS Inc., Chicago, Il, USA).

## Results

### Lifestyle and relative humidity

The prevalence of diabetes was 27% for the entire cohort. No significant variations were observed between islands. When comparing data from participants living in high humidity areas vs. participants living in low humidity ones, the prevalence of diabetes was 42% and 23%, respectively. Specifically, participants 65 to 80 years old living in high humidity areas, had almost double prevalence of diabetes compared with those living in low humidity areas (41% vs. 24%, p = 0.001). In the next age group (i.e., over 80 years old) the percentage increased for participants living in high humidity areas while for those living in low humidity areas remained at the same levels (53% vs. 24%, p = 0.01). Demographic, behavioral and lifestyle characteristics of the sample, by relative humidity, are summarized in Table 1. Compared with elders living in low humidity areas, those living in areas with high humidity were less physically active (p = 0.001), had higher annual financial income (p = 0.005) while had similar education status and smoking habits. Moreover, no differences were observed between elders living in areas with low and high humidity with regards to the level of adherence to the Mediterranean diet.

**Table 1:** Demographic, behavioral and lifestyle characteristics of the MEDIS study participants by climate variations, i.e., low or high relative humidity

	**Low relative humidity**	**High relative humidity**	***p***^***†***^
N	865 (66%)	444 (34%)	
Age (mean ± SD)	74 ± 7.0	73 ± 7.0	0.001
School years (mean ± SD)	6.77 ± 3.6	6.15 ± 3.5	0.75
Annual income >8,000 euro (%)	18	23	0.005
Current smoker (%)	21	18	0.21
Former smoker (%)	39	40	0.75
Minimally or HEPA active (%)	47	35	<0.001
MedDietScore (0–55)	31.7 ± 5	31.3 ± 5	0.27
Living alone (%)	25	23	0.49
Mean temperature (°C)	18.4 ± 0.94	17.1 ± 0.96	<0.001

^*†*^p-values derived using Student’s t-test for the normally distributed variables (i.e., age, school years, MedDietScore), or Pearson’s chi-square test for the categorical ones (i.e., annual income >8000€, current smoker, former smoker, minimally or HEPA active, living alone). HEPA active: health enhancing physical activity.

### Clinical characteristics and humidity

The clinical characteristics and the prevalence of diabetes, based on humidity conditions, are presented in Table 2. As it can be seen, elders living in high humidity areas tended to be more hypertensive (p = 0.07) and more central obese (p = 0.07). Furthermore, they had increased systolic blood pressure rates (p < 0.001), while at the same time they had lower prevalence of hypercholesterolemia (p < 0.001) when compared with the older inhabitants of the low humidity areas. The prevalence of diabetes (p < 0.001) and fasting glucose levels (p < 0.001), were higher in high humidity areas, when compared with the low humidity ones.

**Table 2:** Clinical characteristics and prevalence of the diabetes, of the MEDIS study participants by climate variations, i.e., low or high relative humidity

	**Low relative humidity**	**High relative humidity**	***p***^***†***^
N	865 (66%)	444 (34%)	
Body mass index (mean ± SD kg/m^2^)	28.3 ± 4.4	27.9 ± 4.2	0.13
Central fat (%)	81	88	0.07
Obesity (%)	31	27	0.20
Hypertension (%)	59	64	0.07
Systolic blood pressure (mmHg)	134 ± 15.4	142 ± 19.5	<0.001
Diastolic blood pressure (mmHg)	78 ± 11.5	79 ± 9.7	0.09
Diabetes (%)	24	42	<0.001
Blood glucose levels (md/dL)	115 ± 37	137 ± 59	<0.001
Hypercholesterolemia (%)	53	39	<0.001

^*†*^p-values derived using Student’s t-test for the normally distributed variables (i.e., body mass index, systolic and diastolic blood pressure, fasting blood glucose levels, total cholesterol levels), or Pearson’s chi-square test (i.e., central fat, obesity, hypertension, hyperglycemia, hypercholesterolemia).

Furthermore, an inverse correlation was observed between high humidity areas of living and the physical activity level (rho = - 0.12, p < 0.001). An inverse association was also observed between humidity and adherence to the Mediterranean dietary pattern (rho = - 0.28, p < 0.001). Both findings suggest that people living in high humidity areas tend to be less physically active and away from the Mediterranean dietary pattern.

### Multi-adjusted analysis of diabetes prevalence and climate variations

Age, sex and mean air temperature adjusted logistic regression analysis showed that people living in high humidity areas were 3.1-times more likely to have diabetes, compared to those living in low humidity areas (95% CI 1.65 to 5.94). Specifically, when the relative humidity in an area increases by one degree, the likelihood of an elder having diabetes increases by 12% (odds ratio per 1 degree = 1.12, 95% CI 1.05 to 1.20). However, residual confounding may exists, and therefore multiple logistic analyses were further applied. Results have been displayed in Table 3. A consistent relationship between higher humidity and the likelihood of an elder having diabetes was observed (*models 1, 2 and 3*), even after adjusting for several confounders, such as physical activity, adherence to the Mediterranean diet, smoking habits, living conditions (*model 2*), hypertension, hypercholesterolemia and obesity (*model 3*).

**Table 3:** Results from additive multiple logistic regression models performed to evaluate the association of various bio-clinical, lifestyle characteristics and area’s climate variation of the MEDIS study participants in relation to diabetes mellitus

	***Model 1***		***Model 2***		***Model 3***	
	**OR**	**95% CI**	**OR**	**95% CI**	**OR**	**95% CI**
Age (per 1 year)	0.98	0.95-1.01	0.98	0.94-1.01	0.99	0.95-1.02
Sex (men vs. women)	0.98	0.65-1.47	1.06	0.65-1.72	1.01	0.61-1.06
Mean temperature (per 1°C)	0.95	0.79-1.15	0.95	0.73-1.22	0.97	0.74-1.26
Relative humidity (per 1%)	1.12	1.05-1.20	1.12	1.03-1.22	1.13	1.03-1.24
Living alone (Y/N)	-	-	1.29	0.78-2.12	1.15	0.68-1.93
Physical activity (Y/N)	-	-	0.66	0.41-1.04	0.73	0.46-1.17
MedDietScore (per 1/55)	-	-	0.99	0.94-1.04	0.99	0.95-1.05
Current smoking (Y/N)	-	-	1.11	0.59-2.09	1.23	0.64-2.37
Obesity (Y/N)	-	-	-	-	1.34	0.84-2.14
Hypertension (Y/N)	-	-	-	-	1.84	1.09-3.10
Hypercholesterolemia (Y/N)					0.80	0.48-1.32

OR: odds ratio, CI: confidence interval.

In addition, when data analysis was repeated separately for those between 65–80 years old (*n* = 341), and those above 80 years (*n* = 76), it was found that the positive association of relative humidity with diabetes was significant only in the group of very old people (odds ratio = 1.42, 95% CI 1.10, 1.83), while among those 65–80 years no significant association was found (odds ratio = 1.08, 95% CI 0.98, 1.20). Moreover, when the analysis was stratified by the level of adherence to the Mediterranean diet according to the MedDietScore cutoffs, it was observed that relative humidity was associated with higher likelihood of diabetes, only for the low adherence group (odds ratio = 1.20, 95% CI 1.05, 1.36). For the moderate/high adherence group, relative humidity was not associated with diabetes (odds ratio = 0.98, 95% CI 0.84, 1.16). Moreover, no association was observed between mean temperature and diabetes among those <80 or those >80 years, as well as those close or away to the Mediterranean dietary pattern.

## Discussion

The present work revealed a positive association between climatological parameters and the prevalence of diabetes mellitus; particularly, elder residents of areas with high relative humidity had almost double rates of diabetes than those living in low relative environmental humidity areas. In addition, elders living in areas with high relative humidity had higher systolic blood pressure, lower physical activity and were more likely to have central obesity. The aforementioned relationships, especially among elderly populations, have rarely been reported before.

The prevalence of diabetes in the elderly insular sample of MEDIS study (i.e., 27%) was similar to other recent studies conducted in European countries [30] and in Greece [10,11]. However, the higher prevalence of diabetes in areas with high relative humidity, compared with the low humidity ones, has never been reported before, in similar population samples.

To further explore the aforementioned finding, additional analysis of clinical and lifestyle components and the area’s relative humidity was performed. It was also observed higher prevalence of hypertension, central fat, along with lower physical activity in the elders living in high humidity areas, compared those living in low humidity ones. These factors could play a moderating role in the investigated research hypothesis. Despite the lack of previous studies on relative humidity and prevalence of diabetes and the other aforementioned clinical conditions, a number of studies have already investigated the effect of climate variations (i.e., relative humidity, temperature etc.) on human health [18-20,22]. Specifically, variations in environmental temperature and relative humidity have been associated with deaths from myocardial infarction [17] and hospital admissions for acute coronary syndromes [18,22].

According to Köppen’s climate classification the Mediterranean climate is a particular variety of the subtropical climate found around the Mediterranean Sea. The climate in this area is characterized by relatively warm to hot, dry summers, mild to cool, rainy winters and extended sunshine almost all the year. The islands included in the MEDIS study have Mediterranean climate temperatures, with high air relative humidity and long, hot summers. In the mainland, the mean humidity is around 65% while in the islands is around 75% [31]. High air humidity affects human comfort [32]. Whether these climatological characteristics may promote or not diabetes development is hard to be answered here.

Moreover, several studies have proposed that higher rates of relative humidity are associated with lower mood scores [33,34]. Discomfort and depressive symptoms are associated with lower physical activity unhealthier dietary habits [35,36], conditions that may directly affect the prevalence of diabetes, especially in the group of frail elders. These findings, in combination with observations that were used in the present analysis (i.e., higher environmental humidity correlated with lower physical activity and lower adherence to the Mediterranean diet), may explain the accumulation of these clinical risk factors among individuals living in areas with high relative humidity. However, and before conclusions can be made, it should be taken into account that diabetes mellitus is strongly influenced by various clinical and lifestyle conditions (i.e., hypertension, central fat, physical activity etc.) [12].

Additional analysis showed a consistent association between relative humidity and the likelihood of an elder having diabetes, only in the group of 80 years old and above, and only in elders with low adherence to the Mediterranean diet. The first could be attribute to the fact that prolonged age accumulate several harmful factors, including the additive effect of lifelong living in an environment with high humidity. Regarding the second finding, recent studies have associated high adherence to the Mediterranean diet with better glucose homeostasis in the human body. The Mediterranean dietary pattern emphasizes on a consumption of high monounsaturated fats, mainly from olive oil, and encourages consumption of fruits, vegetables, tree nuts, legumes, whole grains, fish and poultry in low to moderate amounts, a relatively low consumption of red meat, as well as a moderate consumption of alcohol. The proposed mechanism was through regulations in plasma glucose and insulin levels [37]. This effect of a healthy diet in glucose levels along with the fact that progression of aging is followed by accumulation of several clinical and lifestyle (i.e., lower physical activity) cardio metabolic factors [12] may explain the former findings, i.e., the harmful influence of humidity on glucose control among people that are away from the Mediterranean dietary pattern.

Physical activity and adherence to the Mediterranean diet have been associated with insulin resistance and the development of diabetes in many previous studies [12,37]. The current findings also suggested that relative humidity was associated with the aforementioned lifestyle factors, leading to unhealthier lifestyle habits and, consequently, to higher likelihood of having diabetes. Furthermore, the observed twofold prevalence of diabetes mellitus and higher central fat and systolic arterial pressure in the residents of high relative humidity areas may enhance the previous findings and is in accordance with the previously reported association between acute coronary syndromes and relative humidity [18,21].

### Strengths and limitations

The present study has several strengths. It is one of the first to evaluate the effect of climate variations in the health status of a large sample of “healthy”, free-living elderly people in the Mediterranean islands. The limitation of the study is mainly caused by its cross-sectional design and therefore the lack of causal relationships. Also, measuring climate condition only by environmental humidity (and not indoor) can be considered as a study limitation.

## Conclusion

The prevalence of diabetes was high in the studied elderly and free of cardiovascular disease, insular population, and elders living in high humidity areas had almost double prevalence of diabetes, as compared with those living in low humidity residences. The latter attempted to be partially explained by the accumulation of cardiovascular disease factors and by the modifications in lifestyle habits, this population appeared to have made. However, further research is needed to confirm this observation and to understand the underlying mechanisms.

## Competing interests

The authors declare that there are not any financial or non-financial competing interests to declare in relation to this manuscript.

## Authors’ contributions

ST, DP, CH, CL, EP conceived of the study, carried out its designing and implementation, drafted the manuscript, and performed the statistical analysis. MM, KK, NT, AZ, EG, GM, VB participated in the design of the study and revised the manuscript. All authors read and approved the final manuscript.
